# Enhancing Alkaline Biodiesel Transesterification Using Protic Ionic Liquid [DETA][Hx] as a Low-Concentration Additive

**DOI:** 10.1021/acsomega.6c08386

**Published:** 2026-10-14

**Authors:** Monique E. J. Trindade, Diogo S. N. Cardoso, Kleberson R. O. Pereira, Silvana Mattedi, George Simonelli, Luiz C. L. Santos

**Affiliations:** † Oil, Gas, and Biofuels Research Laboratory (PGBio), Postgraduate Program in Chemical Engineering (PPEQ), 28111 Federal University of Bahia (UFBA), Rua Prof. Aristides Novis 02, Federação 40210-630, Salvador/BA, Brazil; ‡ Postgraduate Program in Geochemistry: Petroleum and Environment (POSPETRO), Federal University of Bahia (UFBA), Av. Adhemar de Barros, Ondina 40170-020, Salvador/BA, Brazil; § Chemical Engineering Graduate Program (PPEQ UFBA/UNIFACS), Federal University of Bahia (UFBA), Rua Prof. Aristides Novis 02, Federação 40210-630, Salvador/BA, Brazil

## Abstract

Protic ionic liquids (PILs) have emerged as cost-effective alternatives to aprotic ionic liquids in biodiesel production. In biphasic transesterification systems, the intrinsic immiscibility between methanol and oil phases represents an important limitation affecting reaction performance. In this context, the present study investigates the influence of diethylenetriammonium hexanoate ([DETA]­[Hx]) as a low-concentration physicochemical additive in conventional KOH-catalyzed soybean oil transesterification. A factorial experimental design was employed to evaluate the effects of ionic liquid concentration (0–3% w/w) and reaction time (30–60 min) on biodiesel yield. The results showed that low concentrations of [DETA]­[Hx] significantly affected process performance, increasing biodiesel yield by 5.1% and reaching a gravimetric biodiesel yield of 97.1%. Physicochemical analyses revealed changes in surface tension behavior in systems containing [DETA]­[Hx], which, together with the observed yield response, are consistent with a possible influence of the ionic liquid on physicochemical and interfacial interactions within the reaction medium. FT-IR analyses suggested that the ionic liquid structure remained preserved under the evaluated conditions, supporting the interpretation that [DETA]­[Hx] primarily influences the reaction medium through physicochemical interactions rather than through direct chemical participation in the transesterification reaction. Although direct transport-related measurements were beyond the scope of the present study, the observed behavior suggests that low concentrations of protic ionic liquids may influence phase interactions and reaction performance in alkaline biodiesel transesterification systems. Overall, the present work provides experimental evidence supporting the investigation of protic ionic liquids as low-concentration physicochemical additives for biodiesel production.

## Graphical abstract

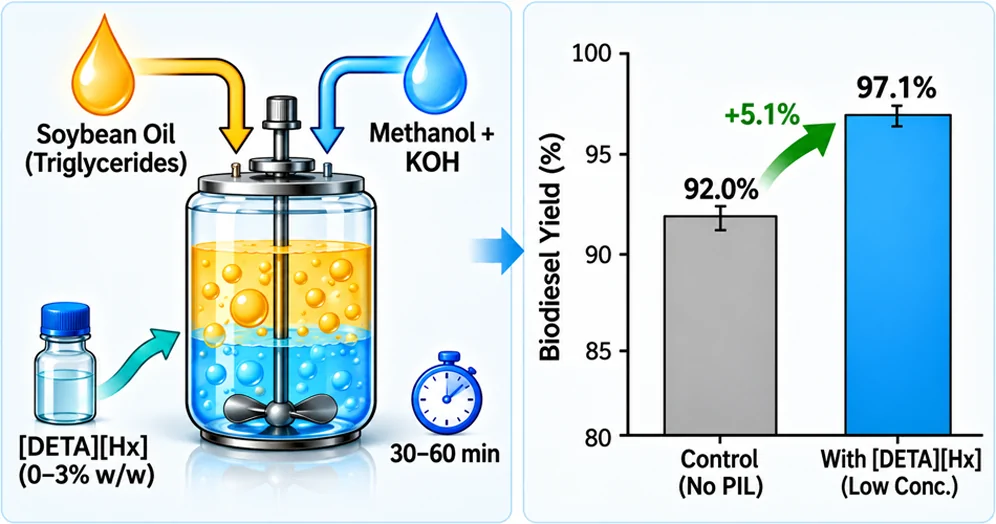

## 1 Introduction

The transesterification of vegetable oils into biodiesel is a well-established route for the production of renewable fuels.^1–5^ Despite its technological maturity, the overall efficiency of alkaline transesterification remains strongly influenced by mass transfer limitations arising from the intrinsic immiscibility between methanol and triglyceride phases.^6–8^ In such biphasic systems, the reaction initially occurs at the liquid–liquid interface, and the apparent reaction rate may therefore depend not only on catalytic activity but also on phase behavior, interfacial transport, and the extent of contact between immiscible reactants.^4,6,9,10^

To mitigate these diffusional limitations, several strategies have been explored to improve interactions between the methanol and oil phases.^5,10,11^ Among them, conventional cosolvents such as tetrahydrofuran, acetone, and diethyl ether have been employed to increase mutual miscibility and improve apparent transesterification performance, with reported biodiesel yields of approximately 97%–98% under optimized conditions.^7,10,12,13^ However, their application may involve volatility, flammability, solvent losses, additional recovery requirements, and increased separation complexity.^7,12^ These limitations motivate the investigation of alternative physicochemical additives capable of influencing biphasic reaction behavior while preserving process simplicity.^14,15^

Unlike conventional molecular solvents, ionic liquids may influence transesterification systems through more complex physicochemical mechanisms involving polarity modulation, amphiphilic organization, hydrogen-bonding networks, and interfacial effects.^7,16–20^ Due to their tunable molecular structure, these compounds may alter solvent organization and phase interactions even at relatively low concentrations, potentially affecting reaction performance without requiring complete phase homogenization.^6,14,21^ From a practical standpoint, conventional solvents benefit from low viscosity and ease of mixing, but their volatility and recovery requirements may compromise process sustainability.^4,7,12,22^ Ionic liquids exhibit negligible vapor pressure and reduced solvent losses, although their higher viscosity, variable toxicity, and cost must be carefully considered for large-scale applications.^4,16,22^ Consequently, their potential applicability extends beyond physicochemical modification of phase interactions and includes the possibility of operating at low additive concentrations without substantial changes to conventional process configurations.

Within this broader class, aprotic ionic liquids (AILs) and related organic salt-based systems have achieved high biodiesel conversions, frequently around 98%, using compounds such as [TBA]­[Arg], [Cho]­[Arg], and [N_2222_]­[Arg].^23–25^ However, these performances have generally been obtained at temperatures of approximately 81–100 °C and under optimized catalyst-loading and alcohol-to-oil conditions.^8,18^ Thus, although these studies demonstrate the effectiveness of ionic-liquid-based systems, the contribution of the ionic liquid itself may be difficult to distinguish from the effects of process intensification.^17,26^

More recently, protic ionic liquids (PILs) have attracted attention because of their relatively simple synthesis, potentially lower cost, and hydrogen-bonding capability.^14,18^ These characteristics make PILs particularly interesting for investigating physicochemical effects on molecular organization and phase interactions, including at relatively low additive concentrations.^6,19^ High biodiesel performance has been reported for PIL-based systems, with conversions generally exceeding 90% and, in several cases, approaching 98%.^19,26,27^ However, these results encompass markedly different process configurations, including temperatures up to 115 °C, relatively high catalyst loadings and alcohol ratios, prolonged reaction times, and microwave- or ultrasound-assisted operation.^19,26,27^ Consequently, although these studies establish the effectiveness of PIL-based approaches, the specific contribution of the ionic liquid may remain difficult to isolate from catalytic and process-intensification effects.^20,28,29^

Despite the broad use of ionic liquids in biodiesel production, their application as low-concentration physicochemical additives, rather than as catalysts or bulk reaction media, remains comparatively underexplored.^15,29^ Recent studies nevertheless indicate that relatively small ionic-liquid additions can produce measurable effects across different biodiesel-related applications, including enzymatic activity, transesterification and esterification performance, and fuel physicochemical properties.^30–34^ These findings demonstrate the broader potential of ionic liquids as functional modifiers; however, their specific use as physicochemical additives in conventional KOH-catalyzed oil transesterification remains insufficiently investigated.^5,14^ Accordingly, the present study addresses this gap by investigating [DETA]­[Hx] not as a catalyst or bulk reaction medium but as a low-concentration physicochemical additive introduced into an otherwise conventional KOH-catalyzed transesterification system.

On the basis of this rationale, the present study investigates the influence of the protic ionic liquid diethylenetriammonium hexanoate ([DETA]­[Hx]) on conventional KOH-catalyzed soybean oil transesterification at concentrations up to 3 wt %. The effects of [DETA]­[Hx] concentration and reaction time on gravimetric biodiesel yield were evaluated together with surface tension and structural characterization analyses to examine the physicochemical behavior of the additive within the reaction system. By maintaining KOH as the alkaline catalyst while investigating [DETA]­[Hx] as a distinct physicochemical additive, this study provides an experimental basis for assessing low-concentration PILs as functional additives in the biphasic biodiesel production.

## 2 Materials and Methods

For laboratory-scale biodiesel production, soya soybean oil, methanol (Synth, 99.8%), and potassium hydroxide (Sigma-Aldrich, 85%) were used. To synthesize the protic ionic liquid diethylenetriammonium hexanoate ([DETA]­[Hx]), the materials used were diethylenetriamine (Sigma-Aldrich, 99%) and hexanoic acid (Merck, 98%).

### 2.1 Soybean Oil Characterization

Tests were carried out for a physical–chemical characterization of soybean oil: acid value, humidity, kinematic viscosity at 40 °C, and specific gravity at 20 °C. The acid value and humidity are the main parameters that guarantee the quality and good preservation of vegetable oils. The acid value was determined by volumetric titration, based on the ABNT NBR 11115 standard.^35^ The humidity level was determined by the gravimetric method, based on the procedure of ANVISA.^36^ Specific gravity and kinematic viscosity analysis were developed using an SVM 3000 Viscometer, based on ASTM D4052 ^37^ and ASTM D445,^38^ respectively.

### 2.2 Synthesis of the Ionic Liquid

[DETA]­[Hx] was synthesized by acid–base neutralization between diethylenetriamine and hexanoic acid. Aliquots of hexanoic acid were slowly added to diethylenetriamine under constant stirring at 300 rpm. The reaction temperature was maintained at approximately 40 °C due to the exothermic nature of the neutralization reaction. Figure 1 illustrates the reaction employed for [DETA]­[Hx] synthesis, in which 3 mol of hexanoic acid is required per mole of diethylenetriamine.

**Figure 1:**
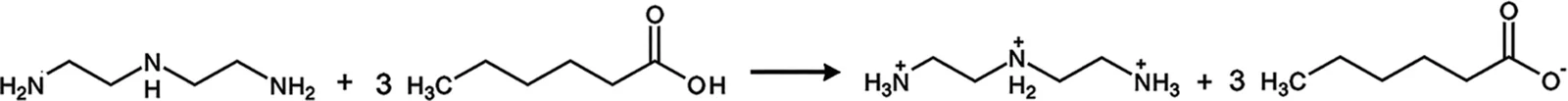
Acid–base neutralization reaction to synthesize diethylenetriammonium hexanoate.

### 2.3 Ionic Liquid Characterization

Specific gravity and kinematic viscosity of [DETA]­[Hx] were obtained through an SVM 3000 viscometer, based on ASTM D4052 ^37^ and ASTM D445,^38^ respectively. Surface tension was analyzed in a Kruss K20 tensiometer by an adapted ring method based on ASTM D971.^39^ The water content was verified using a volumetric Karl Fischer V20 KF Titrator (Mettler Toledo).

To analyze the structure of [DETA]­[Hx], the technique of infrared spectroscopy (FT–IR) was applied, using an infrared spectrophotometer with an IRPrestige-21 Shimadzu Fourier transform. The infrared spectrum of the ionic liquid was obtained in the region of 4000 to 400 cm^–1^ and analyzed in KBr pellets. To confirm the structure of the ionic liquid formed, ^1^H and ^13^C nuclear magnetic resonance (NMR) spectra were recorded by a Bruker Avance 400 MHz spectrometer, with a magnetic field of 9.39 T. For carbon spectra, an external capillary containing CDCl_3_ was introduced coaxially in the NMR tube, and proton spectra were done in solution using CDCl_3_ as solvent; in this case, deuterated 3-(trimethylsilyl) propionic acid-*d*4 (TSP) was also introduced to calibrate the signal at 0 ppm. MestReNova 11.0 software was used to process the obtained data.

### 2.4 Experimental Procedures to Produce Biodiesel

Transesterification reactions were performed on a bench scale in a flat-bottom flask system associated with a reflux condenser supported on stirring and a heating plate. First, 100 g of soybean oil was placed in a flat bottom flask. Separately, the mixture to be added to the oil was prepared. [DETA]­[Hx] was weighed at the proportions of 1, 2, and 3% relative to the mass of oil and 1.5% w/w KOH; these were added at the molar ratio MeOH/oil of 9:1. The reactions were conducted for 30 and 60 min at 60 °C under constant stirring at 600 rpm. For the central point, replicates were run using 1.5% w/w of ionic liquid and 45 min of reaction to obtain an estimate of the pure error. Samples without the addition of [DETA]­[Hx] were also transesterified. After completion of the reaction, the product was transferred to a separatory funnel and allowed to stand for 24 h to ensure complete phase separation prior to purification and characterization steps. Then, glycerol was separated from the biodiesel and four washing cycles were performed with distilled water at 80 °C to remove the remaining alcohol, catalyst, ionic liquid, and glycerol. Thereafter, the biodiesel produced was dried in the oven at 105 °C for a period of 3 h. Finally, the mass yield calculation was performed from eq 1 based on the gravimetric method.^40–42^

1 $$Yield\left(\%\right)=\frac{Biodiesel\;weight\left(g\right)}{Oil\;weight\left(g\right)}\times 100$$

### 2.5 Experimental Design of Biodiesel Production

The transesterification process was carried out through factorial design with 2 independent variables to examine the effects caused by the variation of ionic mass concentration (*X*_1_) and reaction time (*X*_2_) in biodiesel yield (*Y*). The experimental design consisted of 7 trials (4 factorial combinations and 3 replications at the central point) performed at random to avoid systematic errors. The coded intervals and levels of the factors analyzed are listed in Table 1.

**Table 1:** Coded Variables of the Experimental Design

independent variables	symbol	levels and ranges		
		–1	0	+1
ionic liquid concentration (% w/w)	*X*_1_	0.0	1.5	3.0
time of reaction (min)	*X*_2_	30	45	60

According to Table 1, the values +1 and −1 represent the upper and lower levels, respectively, studied for both variables. Zero (0) corresponds to the central point of the variable, executed with replicates for internal estimation of the pure error.^40,41^ The complete model of biodiesel production that correlates the product yield with the studied process variables was established from linear regression of data (eq 2). *Y* refers to response (biodiesel yield), β_0_ is the intercept, β_1_ and β_2_ are the linear terms, and β_3_ is the interaction term, model parameters that are estimated, X_1_ and X_2_ are the coded independent variables.^40,43^

2 $$Y=\beta_{0}+\beta_{1}X_{1}+\beta_{2}X_{2}+\beta_{3}X_{1}X_{2}$$

The analytical steps performed included analysis of variance (ANOVA), regression evaluation, and response surface graph of the interaction effects of the factors to identify the experimental conditions associated with higher biodiesel yield. The quality of the fit of the polynomial model equation was evaluated by the coefficient of determination *R* ^2^. The p-value was used as a basis for measuring the significance of regression coefficients; values less than 0.05 express that the coefficient is relevant, otherwise, it is insignificant for the model.^19,40,42^

### 2.6 Biodiesel Characterization

To determine the quality of the biodiesel produced, the samples were characterized by the analysis of specific gravity at 20 °C and kinematic viscosity at 40 °C, using Anton Paar Viscometer SVM 3000, based on ASTM methods D4052 ^37^ and ASTM D445,^38^ respectively. A visual inspection was performed to check the appearance of biodiesel and to check whether it was clear and free of impurities. Flash point analysis was also performed using Petrotest PM4 equipment based on the ASTM D93 method.^44^

With the purpose to define the properties of biodiesel produced, the results of the tests carried out were compared to European Standard EN 14214,^45^ American Standard ASTM D6751,^46^ and Brazilian Standard ANP n° 45/2014,^47^ which specify the parameters that guarantee the quality of biodiesel for commercialization.

The experimental methodology was designed to evaluate the effect of [DETA]­[Hx] addition on the biodiesel production performance and selected physicochemical properties of the reaction system. Direct transport-related parameters, such as the volumetric mass transfer coefficient (kLa), interfacial area, and droplet size distribution, were not experimentally determined. Therefore, the experimental approach adopted in this study was intended to evaluate the reaction performance and selected physicochemical responses associated with [DETA]­[Hx] addition, without constituting a direct measurement of transport phenomena.

## 3 Results and Discussion

### 3.1 Ionic Liquid Characterization

From the ionic liquid characterization analysis, diethylenetriammonium hexanoate [DETA]­[Hx] showed the following properties at 60 °C: density of 974.5 kg.m^–3^, kinematic viscosity of 388.72 mm^2^.s^–1^, surface tension of 24.3 mN·m^–1^, and water content of 1.0 ± 0.1% (w/w). Ionic liquids constantly absorb water even after its production due to its hygroscopic characteristic.

The physicochemical behavior observed in the present study is also consistent with previous independent measurements reported for [DETA]­[Hx] by Ferreira et al.^14^ In that study, [DETA]­[Hx] exhibited a critical micelle concentration of 235 ppm at 60 °C and an interfacial tension of 11.8 ± 0.4 mN·m^–1^ at 1.5 wt % in a soybean-oil-containing biphasic system. These independently obtained results provide complementary evidence that surface activity and interfacial behavior have also been observed for [DETA]­[Hx] under related conditions, while the interpretation of the present results remains based on the physicochemical and structural characterization performed directly in this study.

When the main intention is to analyze the thermodynamic properties of ionic liquids, strict control of water content is generally required.^48^ In the present study, [DETA]­[Hx] was employed in its as-synthesized form, without a dedicated postsynthesis drying or purification step, since [DETA]­[Hx] was investigated in its as-synthesized form at relatively low additive concentrations. The water content analysis was performed after completion of the biodiesel production experiments; therefore, the measured value of 1.02 wt % may be partially attributed to moisture absorption from the environment during storage and handling. Although water is known to potentially affect alkaline transesterification systems through triglyceride hydrolysis and soap formation,^4,6,24^ the relatively low water content measured for [DETA]­[Hx] did not prevent the achievement of high biodiesel yields under the evaluated conditions. This behavior suggests that the residual moisture associated with the ionic liquid was not sufficient to significantly suppress transesterification performance or promote severe alkaline side reactions under the operational conditions investigated.

To clarify the potential influence of water introduced by the ionic liquid, the effective water contribution associated with [DETA]­[Hx] was estimated under the highest loading evaluated in this study. Considering the addition of 3.0 g of ionic liquid per 100 g of soybean oil and the measured water content of 1.02 wt %, approximately 0.0306 g of water would be introduced into the reaction medium. This corresponds to about 0.031 wt % relative to the oil mass, which is below the water-content threshold commonly reported for conventional alkaline transesterification systems (∼0.06 wt %),^6,7^ above which hydrolysis, soap formation, catalyst consumption, and separation difficulties become increasingly significant. Therefore, although residual moisture in ionic liquids should be carefully controlled, the amount of water introduced by [DETA]­[Hx] under the evaluated conditions was relatively low and unlikely to severely inhibit alkaline transesterification, which is consistent with the high biodiesel yields obtained in this work. Beyond the moisture-content evaluation, infrared spectroscopy was used to verify the chemical structure of the synthesized ionic liquid. The analysis confirmed the structure of [DETA]­[Hx], as shown in Figure 2.

**Figure 2:**
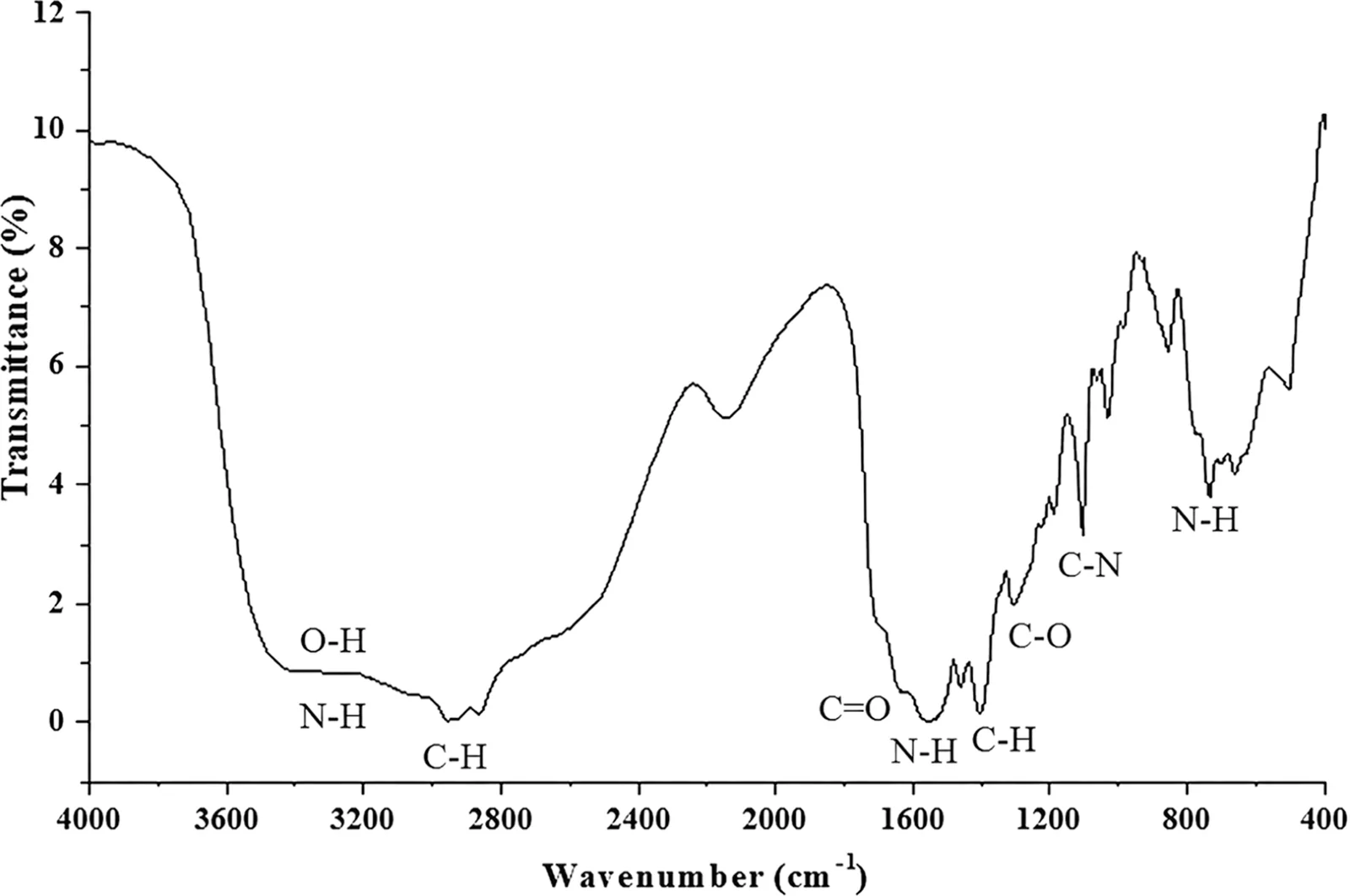
FT-IR of diethylenetriammonium hexanoate [DETA]­[Hx].

To identify the structure of ionic liquid based on ammonium neutralization, the spectrum must present the cation band, corresponding to the N–H structure of primary amines, which is present around 3600–3200 cm^–1^ and reproduced in a broad peak at 1640–1550 cm^–1^. It can still be seen that there is an out-of-plane bending corresponding to N–H in 900–650 cm^–1^, and in addition, C–N stretching is presented in 1350–1000 cm^–1^. The anion peak is visualized by the carbonyl, corresponding to the C=O stretching of the carboxylic acid group, displayed in the range of 1850–1610 cm^–1^ with a large stretching O–H in 3400–2400 cm^–1^. Complex compounds, such as ionic liquids, may present overlaps between the cation and the anion bands. It is important to identify other bands to confirm that different structures are present in the compound. The peak between 3000 and 2800 cm^–1^ and ratified in the range of 1450–1375 cm^–1^ refers to the C–H sp3 bonds of the alkyl chain of the anion and the ethyl cation groups. Bands between 1300 and 1250 cm^–1^ are assigned to the C–O bond of carboxylic acids with medium intensity.^49^ ^1^H NMR and ^13^C NMR spectra were analyzed to confirm the structure of [DETA]­[Hx] and are represented, respectively, in Figure 3(a, b).

**Figure 3:**
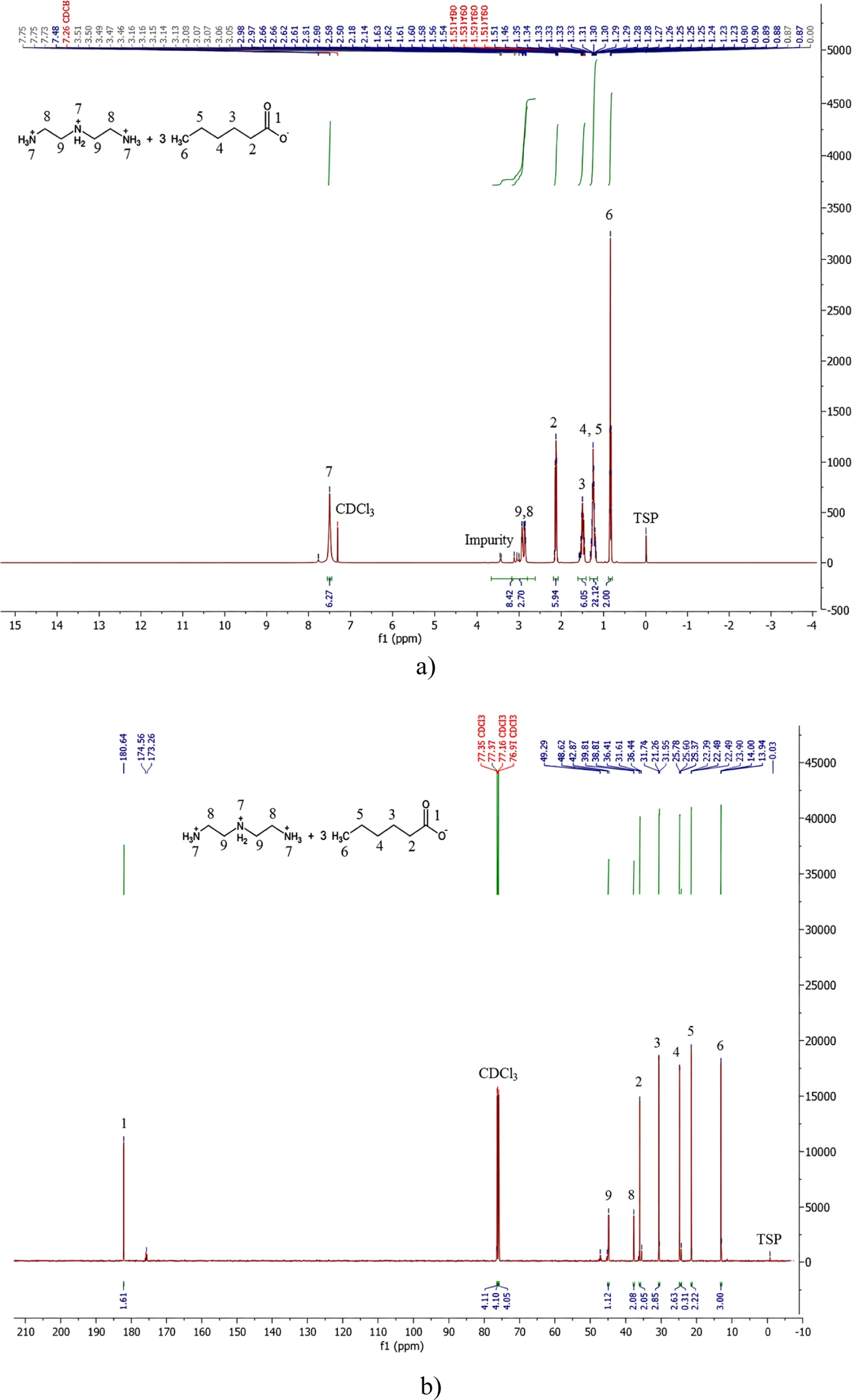
(**a**) ^1^H NMR spectra and line shape analysis for PIL [DETA]­[Hx] and (**b**) ^13^C NMR spectra and line shape analysis for PIL [DETA]­[Hx].

The peaks depicted in the spectra refer to the arrangement of hydrogen and carbon atoms in the ionic liquid structure. ^1^H NMR spectrum is commonly used to assess the compound’s impurities. The peak at 0 ppm refers to TSP and the peak at 7.28 represents the solvent used (CDCl_3_). Because it is a chemical equilibrium reaction that moves more toward product formation than to the reactant, it is susceptible to proton transfer between acid and base, even after the ionic liquid is formed as there is a minimal amount of the reactant in the medium. Therefore, hydrogens for peak 7 are represented at 7.48 ppm and can change positions with each other. All chemical shifts of cation and anion structure were identified and correspond to the suggested synthesized structure. Peaks are reported in Table 2.

**Table 2:** ^1^H and ^13^C NMR with Chemical Shifts (ppm) of PIL [DETA]­[Hx]

peaks	PIL [DETA][Hx]	
	^1^H	^13^C
1	-	180.64
2	2.16	36.41
3	1.54	31.76
4	1.29	25.78
5	1.29	22.49
6	0.88	13.94
7	7.48	-
8	2.89	38.81
9	2.98	48.29

Based on the NMR and FT–IR analysis, it was possible to confirm that [DETA]­[Hx] was formed due to the presence of functional groups and chemical elements that confirms the structure of this ionic liquid. As the temperature used in the biodiesel production experiments (60 °C) may lead to amide formation between the ammonium salt and carboxylate anion, it is important to analyze whether the NMR spectrum presents a signal associated with the amide functional group, which contains a carbonyl (C=O) bonded to nitrogen. When the solvent is chloroform (CDCl_3_), the amide peaks at a displacement above 8.0 ppm in ^1^H NMR.^50^ There is no peak for the amide functional group. Integrating the peaks relative to the –CH_3_ terminal of the carboxylate (peak 1) and comparing with the integral of the intermediate carboxyl peaks (peak 7 and 8), and by verifying the integrality of the largest of the impurity peaks, the ratio of amine to acid is confirmed. Being the excess of acid and the impurity content less than 2%. In this way, the purity of the liquid can be taken as higher than 98%. Considering the water content, the purity is greater than 97%.

### 3.2 Soybean Oil Characterization and Tests of Biodiesel Production

Soya soybean oil was characterized by analysis of acidity index, humidity, specific gravity at 20 °C, and kinematic viscosity at 40 °C. Data presented in Table 3 show the values of the analysis performed. It was observed that the values of these properties agreed with data found in the literature, indicating that soybean oil met the quality standards. The quality of the raw material, soybean oil, is directly related to the quality of the final product, biodiesel. The acid value and moisture content of the oil used in biodiesel production must be low to prevent the hydrolysis of triglycerides during transesterification.^4,51–53^ This avoids the formation of free fatty acids, which are detrimental to alkaline transesterification, as they react with the catalyst to form soap in the reaction medium.^4,6,51,52^

**Table 3:** Soya Soybean Oil Physical–Chemical Characterization

experiments	analysis	reference value	ref.
acid value (mgKOH.g^‑1^)	0.28 ± 0.01	≤0.50	^54^
humidity (%)	0.09 ± 0.03	≤0.10	^12,54^
kinematic viscosity at 40 °C (mm^2^.s^‑1^)	31.06 ± 0.02	25.60–33.00	^38,52,54^
specific gravity at 20 °C (g.cm^‑3^)	0.920 ± 0.001	0.913–0.925	^52,54^

[DETA]­[Hx] concentrations ranging from 0 to 3% (w/w) were evaluated using a methanol-to-oil molar ratio of 9:1, 1.5% (w/w) KOH, and a reaction temperature of 60 °C. Figure 4 illustrates the effect of [DETA]­[Hx] concentration on the gravimetric biodiesel yield at reaction times of 30 and 60 min.

**Figure 4:**
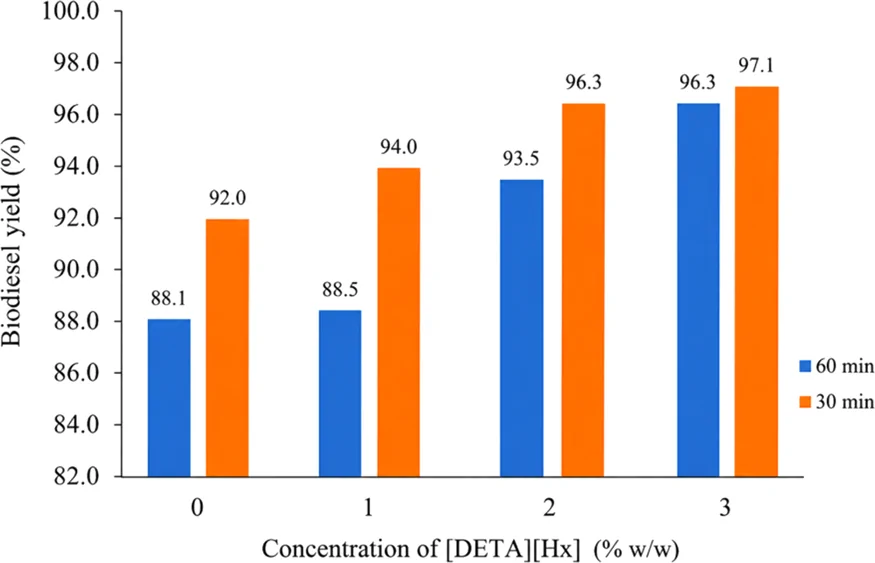
Biodiesel yield with different concentrations of [DETA]­[Hx] in 30 and 60 min of reaction.

At both reaction times, an increase in the [DETA]­[Hx] concentration resulted in higher gravimetric biodiesel yields. At 60 min, the yield increased from 88.1% in the absence of [DETA]­[Hx] to 96.3% at 3% (w/w), corresponding to an increase of 8.2 percentage points. At 30 min, the gravimetric yield increased from 92.0 to 97.1%, corresponding to an increase of 5.1 percentage points. Therefore, although the absolute increase relative to the respective blank was greater at 60 min, the highest gravimetric biodiesel yield was obtained at 30 min with 3% (w/w) [DETA]­[Hx].

In the absence of [DETA]­[Hx], a higher gravimetric yield was already observed at 30 min than that at 60 min. Prolonged reaction times can reduce biodiesel yield through competing phenomena, including the reverse transesterification reaction and soap formation.^4,53^ In the presence of [DETA]­[Hx], the highest yields were also obtained at 30 min. Under the evaluated conditions, this behavior indicates that increasing the reaction time from 30 to 60 min did not provide an additional performance advantage, while the progressive increase in yield with the [DETA]­[Hx] concentration supports a concentration-dependent influence of the additive on reaction performance. Considering the physicochemical properties of [DETA]­[Hx] discussed above, this effect may be associated with modifications in the physicochemical interactions within the biphasic reaction medium.^6,17^

The relatively low concentrations of [DETA]­[Hx] employed under the evaluated conditions may represent a relevant aspect from a process intensification perspective. Measurable biodiesel-yield improvements were obtained using a maximum ionic liquid concentration of only 3 wt % without requiring substantial modifications to the conventional alkaline transesterification configuration. Several ionic-liquid-based biodiesel systems reported in the literature frequently employ elevated ionic liquid concentrations, intensified heating conditions, or catalyst-centered approaches. For example, Wang et al.^25^ employed 20 wt % of [N_2222_]­[Arg] to achieve 98.4% conversion during soybean-oil transesterification. Similarly, Cai et al.^55^ used 12 wt % additive loading for oleic acid conversion, while Wang et al.^56^ employed 20 wt % of a phosphotungstic acid-based ionic liquid supported on magnetic nanocellulose. In contrast, the present study evaluated whether comparatively low concentrations of a protic ionic liquid could influence the reaction performance under moderate operational conditions.

Ionic liquids are generally associated with higher initial costs than conventional cosolvents and homogeneous catalysts, with estimated costs in some cases ranging from two to one hundred times those of conventional solvents.^3,17,18,57^ However, protic ionic liquids may offer economic advantages due to their relatively simple synthesis routes, while recovery and reuse may further reduce the effective ionic liquid cost over successive operational cycles.^17,18^ This potential has been demonstrated in ionic liquid-assisted biodiesel systems, with relatively stable performance reported over multiple cycles. Sangeetha and Baskar^57^ reported that the amphiphilic system [C_16_N-PS]-HPMo-1 maintained 95.86% biodiesel yield after six reaction cycles. Ibrahim et al.^26^ employed [NMP]­[HSO_4_] under microwave-assisted conditions and observed conversion values above 95% over six reuse cycles without detectable structural degradation based on FT-IR analysis. Gautam et al.^34^ also evaluated an ionic-liquid-based nanophotocatalyst (ILNPC) under optimized ultrasound and microwave conditions, reporting 84.27% biodiesel yield after the fifth reuse cycle while preserving structural integrity according to X-ray diffraction and FT-IR analyses.

Additionally, the practical feasibility of ionic-liquid-assisted systems depends strongly on efficient recovery and minimization of ionic liquid losses during purification stages.^18^ Conventional aprotic ionic liquids are often associated with relatively high production costs, frequently exceeding USD 50 kg^–1^.^20^ ^,^ ^58^ ^,^ ^59^ In contrast, certain protic ionic liquids can be produced through simpler acid–base neutralization routes, with estimated costs in the range of USD 0.78–3 kg^–1^.^18^ ^,^ ^20^ ^,^ ^58^ These differences in cost and recovery requirements motivated the preliminary mass-balance sensitivity analysis presented below.

Accordingly, a preliminary mass balance sensitivity analysis was performed using the condition providing the highest gravimetric biodiesel yield in this study. The analysis estimates the maximum [DETA]­[Hx] loss that could be tolerated before the economic value associated with the additional biodiesel produced is completely offset, providing an initial quantitative framework for relating the biodiesel yield enhancement, ionic liquid cost, and recovery requirements.

The maximum tolerable [DETA]­[Hx] loss was estimated from a simplified mass-balance criterion in which the economic value associated with the additional biodiesel produced was assumed to offset the cost of unrecovered ionic liquid. Under this condition, the maximum tolerable loss can be expressed in eq 3.

3 $$L_{máx}=\frac{\Delta m_{BD}\times P_{BD}}{m_{IL}\times C_{IL}}$$

where *L*_máx_ is the maximum tolerable [DETA]­[Hx] loss fraction, Δ*m*_BD_ is the additional biodiesel produced, *P*_BD_ is the biodiesel value, *m*_IL_ is the ionic liquid mass added, and *C*_IL_ is the ionic liquid production cost. The analysis was based on the condition providing the highest gravimetric biodiesel yield among those evaluated in this study, corresponding to an additional 5.1 g of biodiesel and 3.0 g of [DETA]­[Hx] per 100 g of soybean oil processed. For the sensitivity analysis, a biodiesel reference value of USD 1.00 kg^–1^ was adopted. This value is consistent with minimum selling prices reported in previous techno-economic studies, which are approximately USD 0.90 to 1.05 kg^–1^ depending on feedstock, production scale, and process assumptions.^54,57,60^ Therefore, the selected value represents a reasonable reference scenario for evaluating the relationship between biodiesel yield enhancement and ionic liquid recovery requirements. According to eq 3, the maximum tolerable ionic liquid loss corresponds to the loss threshold at which the economic value associated with the additional biodiesel produced equals the cost of the unrecovered ionic liquid. The results obtained from this preliminary mass-balance sensitivity analysis are presented in Figure 5.

**Figure 5:**
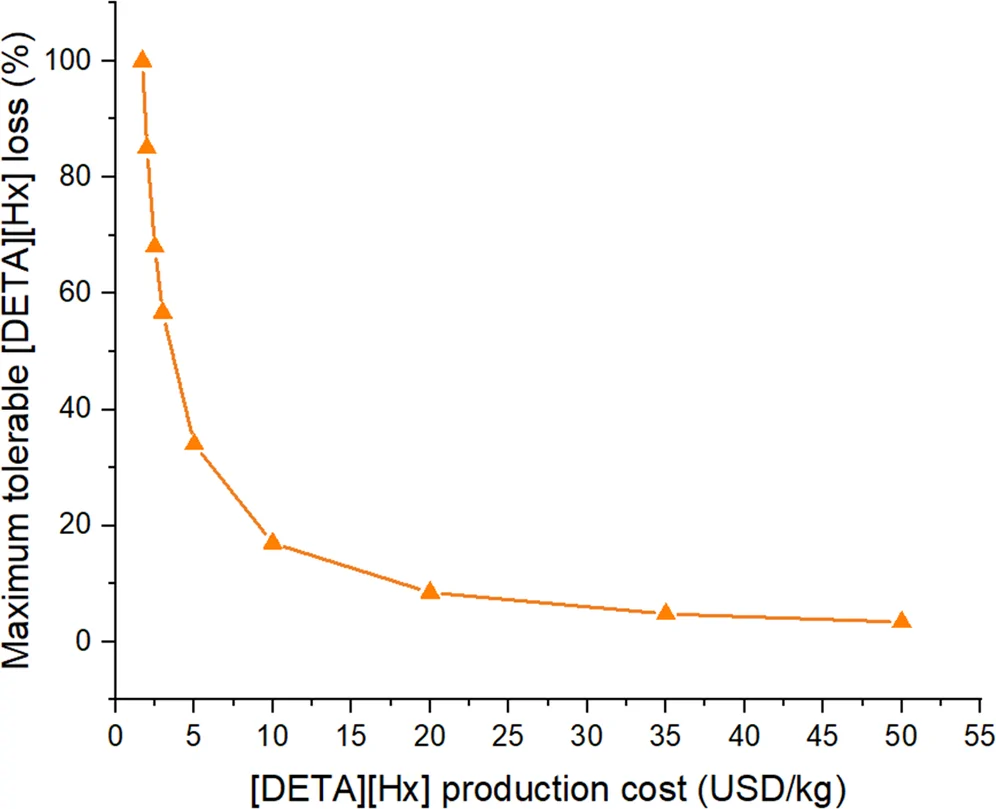
Maximum tolerable [DETA]­[Hx] loss as a function of estimated ionic liquid production cost based on a preliminary mass-balance sensitivity analysis.

The sensitivity analysis indicates that the practical significance of [DETA]­[Hx] is strongly dependent on the ionic liquid cost and recovery efficiency. For estimated [DETA]­[Hx] production costs below approximately USD 1.70 kg^–1^, which corresponds to the break-even cost predicted by eq 3, the economic value associated with the additional biodiesel produced exceeds the cost of the total ionic liquid added under the simplified assumptions adopted. At the upper limit of the reported low-cost PIL range (USD 3.00 kg^–1^), however, the maximum tolerable loss decreases to approximately 56.7%, indicating that recovery efficiency becomes increasingly important for preserving the economic benefit associated with the additional biodiesel produced. For comparison, extending the analysis to ionic liquid costs approaching USD 50 kg^–1^, values commonly reported for several aprotic ionic liquids, reveals a drastic reduction in the tolerable loss threshold, highlighting the potential economic advantage associated with low-cost protic ionic liquids. These results should be interpreted as a preliminary mass balance assessment intended to provide a quantitative perspective on recovery requirements rather than as a complete techno-economic evaluation.

### 3.3 Statistical Analysis

The complete factorial design of 2 independent variables and biodiesel yield is represented in Table 4. The mathematical model obtained by the regression of parameters is shown in eq 4. The positive sign indicates a synergistic effect on the yield increase, and the negative sign denotes an opposite effect.^40^ In the model, *X*_1_ is the concentration of [DETA]­[Hx] (% w/w), *X*_2_ is the reaction time (minutes), and *Y* is the biodiesel yield (%).

4 $$Y=93.95+6.63X_{1}-2.31X_{2}+1.53X_{1}X_{2}$$

**Table 4:** Factorial Design 22 Matrix

standard run	*X*_1_		*X*_2_		*Y*
	ionic liquid concentration (% w/w)	coded	time of reaction (min)	coded	yield (%)
1	0.0	–1	30	–1	92.0
2	3.0	+1	30	–1	97.0
3	0.0	–1	60	+1	88.1
4	3.0	+1	60	+1	96.3
5	1.5	0	45	0	94.1
6	1.5	0	45	0	95.3
7	1.5	0	45	0	94.8

The empirical model was tested with ANOVA at 95% confidence level (Table 5). In ANOVA, the *F*-value is an indicator used to determine the significance of the model. The calculated *F* of the model must be greater than the tabulated *F* value. On the basis of the *F* value evaluation, this model is statistically significant at the 95% confidence level. Calculated *F* was 12.9350 for this model and is 1.39 times higher than the tabulated *F* value. For the lack of fit test, the tabulated *F* value must be greater than the calculated *F* value. In this case, the calculated *F* was 9.8862, being 1.87 times smaller than the tabulated *F*. Therefore, the empirical model of this study was adequate to express the relationship between the response and independent variables.

**Table 5:** Analysis of Variance for the Biodiesel Yield Model

factor	sum of squares (SS)	degrees of freedom (DF)	mean square (MQ)	*F*	*p*-value	F0.05
model	51.6339	3	17.2113	12.9350	<0.0076	9.2766
(*X*_1_) ionic liquid	43.9569	1	43.9569	130.8890	0.0076	
(*X*_2_) time	5.3361	1	5.3361	15.8891	0.0576	
(*X*_1_) by (*X*_2_)	2.3409	1	2.3409	6.9704	0.1185	
residual	3.9918	3	1.3306			
lack of fit	3.3201	1	3.3201	9.8862	0.0880	18.5128
pure error	0.6717	2	0.3358			
total SS	55.6257	6				
*R* ^2^ = 0.9282						

*P*-values are associated with the interaction strengths of each parameter and are used as a tool to verify the importance of each coefficient. The lower the *p*-value, the greater the significance of the corresponding coefficient.^19,24^ In this study, the *p*-value of the model was lower than 0.0076, thus qualifying it as adequate for this experimental design. The *p* value of the lack of fit was 0.0880 and was not relevant to the model. The low value of pure error and residuals indicated a good reproducibility of the experimental data and adequacy of the model to represent the relation between independent variables and response.^26^

The model presented a coefficient of determination (*R* ^2^) of 0.9282, indicating that 92.82% of the observed variability in the gravimetric biodiesel yield was explained by the fitted model within the investigated experimental domain. Together with the nonsignificant lack of fit, this result supports the adequacy of the model for describing the effects of the investigated factors under evaluated conditions.

### 3.4 Effects of Parameter Interaction in Biodiesel Production

The selection of [DETA]­[Hx] concentration and reaction time as the factors investigated in the factorial design was directly related to the specific objective of this study. Rather than performing a global optimization of the conventional transesterification variables, the present work sought to determine whether the introduction of a previously unexplored PIL at low concentration could affect the performance of an otherwise fixed KOH-catalyzed transesterification system. Therefore, [DETA]­[Hx] concentration was selected as the primary experimental factor, while reaction time was included to determine whether the influence of the additive persisted or changed with reaction duration. The remaining operational parameters, including temperature (60 °C), methanol-to-oil molar ratio (9:1), KOH concentration (1.5% w/w), and stirring speed (600 rpm), were maintained constant to provide a common reaction environment for assessing these effects. Figure 6 illustrates the corresponding response surface.

**Figure 6:**
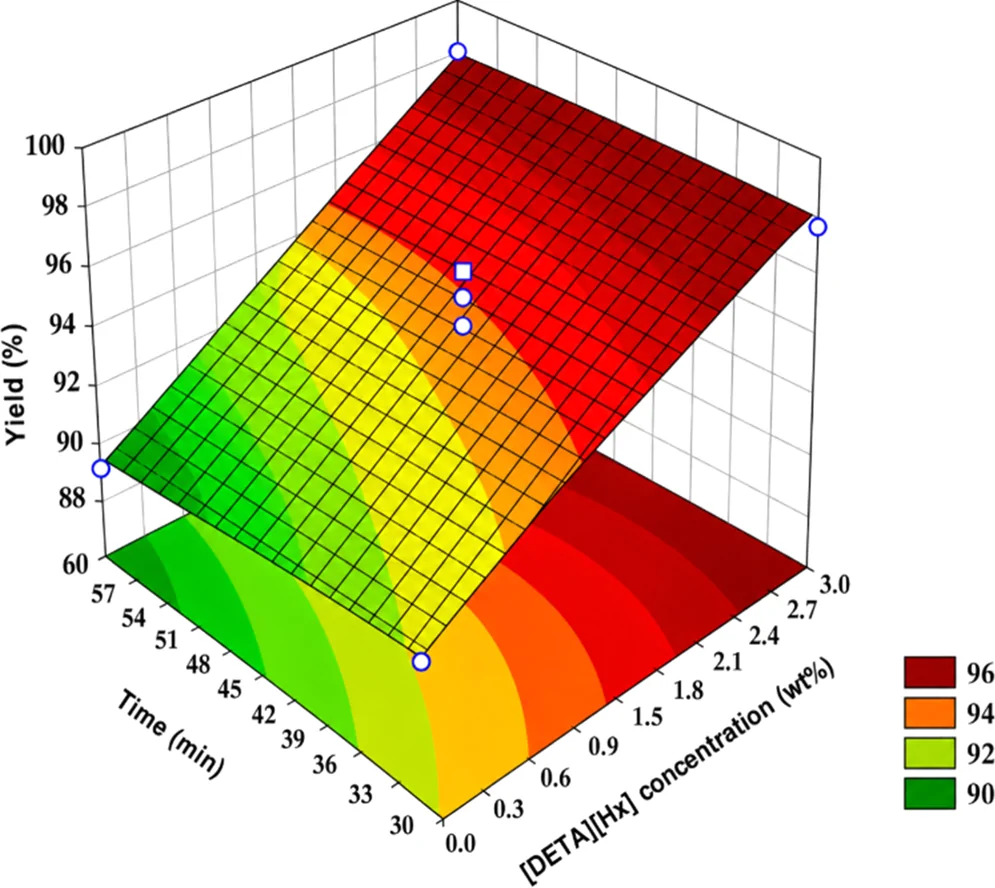
Surface plot of the interaction effect of the ionic liquid concentration and time of reaction.

Increasing the [DETA]­[Hx] concentration positively affected the gravimetric biodiesel yield, reaching approximately 97% at 3% (w/w). Although the fitted model showed a negative coefficient for the reaction time, this factor was not statistically significant at the 95% confidence level (*p* = 0.0576). Likewise, the interaction between the [DETA]­[Hx] concentration and reaction time was not statistically significant (*p* = 0.1185). Within the investigated conditions, the highest gravimetric biodiesel yield was obtained at 3% (w/w) [DETA]­[Hx] and 30 min.

Many cosolvents can be used to increase miscibility among the reactants of oil transesterification in biodiesel. However, the improvement of biodiesel synthesis may be limited due to the physical properties of cosolvents, the reaction operational conditions, and undesirable reactions.^4,26,52^ [DETA]­[Hx] has a relatively low surface tension (24.3 mN m^–1^), a physicochemical characteristic that may contribute to modified interactions between the immiscible oil and methanol phases and, consequently, may be associated with changes in the physicochemical behavior of the biphasic reaction medium.^6,19^

Although pH measurements were not performed in the present study, the acid–base properties of the precursor species provide qualitative insight into the expected behavior of the [DETA]­[Hx]/methanol system. Hexanoic acid exhibits a p*K*_a_ of approximately 4.8, whereas diethylenetriamine is a polyamine with conjugate-acid p*K*_a_ values near 9–10.^14,61^ Therefore, the proton transfer associated with [DETA]­[Hx] formation is expected to generate a predominantly ionic system, whose acid–base behavior in methanol is governed by the resulting ionic species and their interactions with the solvent.^14,23,24^ Under methanolic conditions, this behavior may be further influenced by ion pairing, hydrogen-bonding interactions, and solvent effects.

Potassium hydroxide, a strong base, was employed as the catalyst in the transesterification system. Therefore, FT–IR analysis was used to investigate whether the contact of [DETA]­[Hx] with KOH and methanol resulted in detectable changes in the principal vibrational features of the ionic liquid. For this purpose, the spectra of the individual components were used as spectral references and compared with that of the combined [DETA]­[Hx] + KOH + methanol system, prepared using the same compositional proportions employed in the transesterification experiments (Figure 7). This comparison was specifically intended to assess the structural preservation of [DETA]­[Hx] in the chemical environment preceding its contact with the soybean oil.

**Figure 7:**
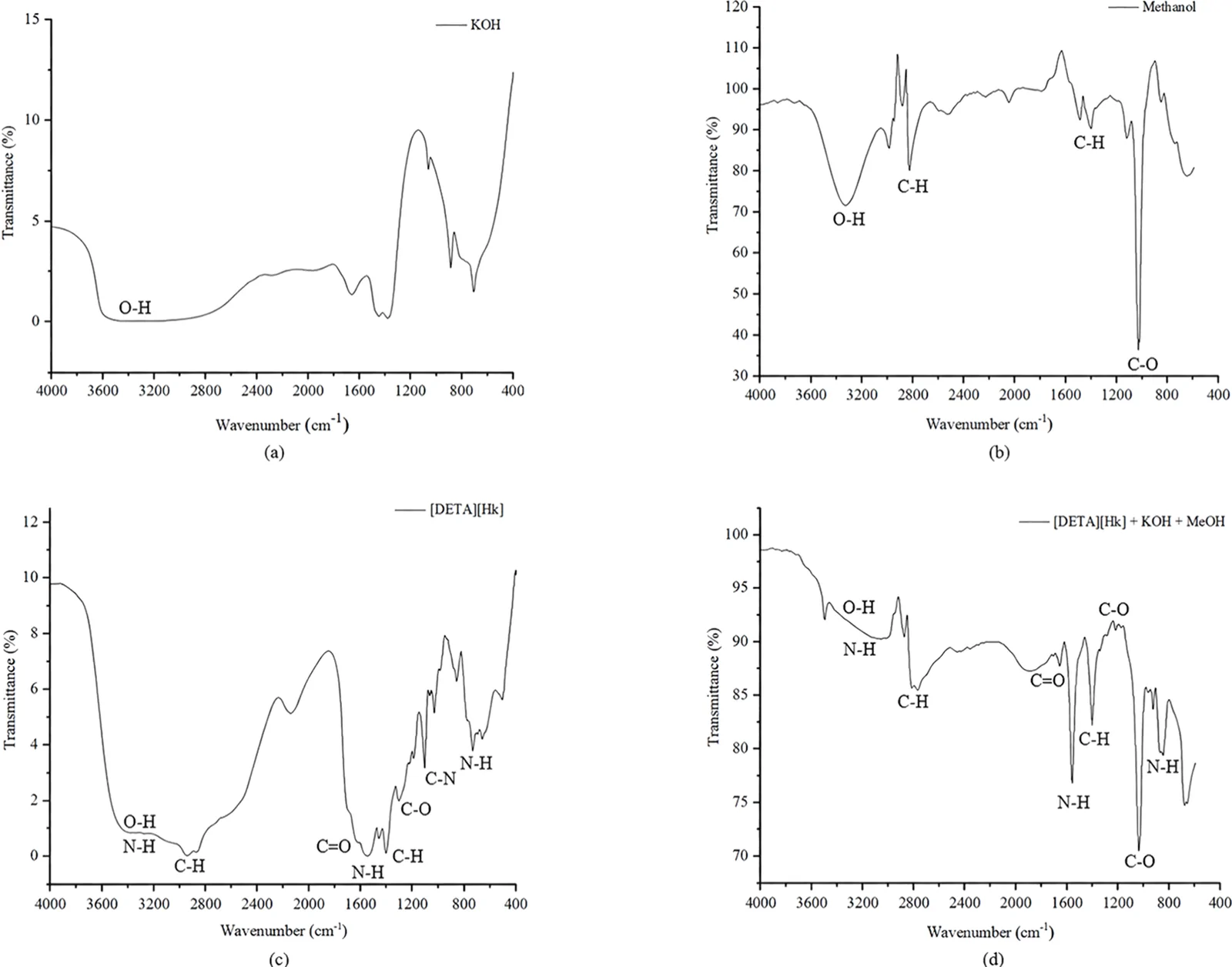
Comparative FT–IR spectra of (**a**) potassium hydroxide (KOH), (**b**) methanol, (**c**) [DETA]­[Hx], and (**d**) the [DETA]­[Hx] + KOH + methanol mixture.

As shown in Figure 7, the characteristic absorption bands of the individual components are identified in spectra (a), (b), and (c), while the spectrum obtained for the combined system in (d) preserves the principal vibrational features associated with [DETA]­[Hx] and methanol. The broad bands observed in the 3600–3200 cm^–1^ region are associated with overlapping N–H and O–H stretching vibrations, whereas the bands attributed to carbonyl groups (1850–1610 cm^–1^), C–O vibrations (1200–1020 cm^–1^), and aliphatic C–H stretching and bending modes (3000–2800 cm^–1^ and 1450–1375 cm^–1^) remain identifiable within the mixed system.

The preservation of the principal vibrational bands after mixing suggests that no major structural transformations occurred under the evaluated conditions. These observations are consistent with the interpretation that [DETA]­[Hx] remains structurally stable within the reaction medium and may influence transesterification behavior primarily through intermolecular and phase interaction effects rather than through direct chemical transformation. In this context, FT–IR analysis provides spectroscopic evidence regarding the structural preservation and physicochemical compatibility of [DETA]­[Hx] within a reactive medium.

Unlike conventional cosolvents, which primarily promote phase homogenization through dilution and mutual solubilization, the behavior observed for [DETA]­[Hx] is consistent with a physicochemical influence involving intermolecular and phase-related effects.^4,7,10,12^ However, direct comparisons should be interpreted cautiously because of differences in composition, operating conditions, and functions performed by these additives in transesterification systems.

Surface tension is temperature-dependent and generally increases as temperature decreases over the range commonly investigated in biodiesel-related systems.^20^ Because surface-active compounds can modify the physicochemical behavior of liquid mixtures, surface tension and specific gravity were evaluated to examine how the addition of [DETA]­[Hx] affected the oil- and methanol-containing systems. The results are presented in Table 6.

**Table 6:** Properties Analyzed at 30 °C for the Mixture [DETA]­[Hx], Alcohol, and Soybean Oil

reactant	surface tension (mN m^‑1^)	specific gravity (g.cm^‑3^)
soybean oil	32.00 ± 0.04	0.91343 ± 0.00001
methanol	21.60 ± 0.05	0.78300 ± 0.00001
[DETA][Hx] + soybean oil	22.40 ± 0.04	0.79726 ± 0.00001
[DETA][Hx] + methanol	28.7 ± 0.7	0.90697 ± 0.00001

On the basis of these data, it is noticed that in the mixture of [DETA]­[Hx] and soybean oil, there is a reduction in the surface tension of the oil (22.40 mN·m^–1^) when compared to the pure oil tension, suggesting that the hydrophobic moieties of [DETA]­[Hx] may interact with the oil phase and contribute to reduced cohesive interactions within the system. Cai et al.^19^ and Alreshidi et al.^18^ reported that the cohesive forces between ionic liquids and long-chain alcohols are strong and generate greater interactions at the interface between them, which may contribute to the reduction of interfacial tension. However, when comparing pure methanol and the mixture of [DETA]­[Hx] and methanol, the opposite occurs, the surface tension increases (28.7 mN·m^–1^).

In binary ILs and methanol systems, the literature reports significant intermolecular interactions, interstitial coupling, electrostatic interactions, dipole bonds, and hydrogen bonds. Ammonium-based ILs have greater interaction with methanol compared to phosphonium-based ones.^17,18^ The observed increase in surface tension may be associated with the complex balance of intermolecular interactions arising from the marked difference between the molecular dimensions of methanol and the ionic species, together with the coexistence of polar active sites and nonpolar regions in [DETA]­[Hx].^6,17^ Since both contain simple bonds and the system is liquid, the free rotational mobility around single bonds of each structure makes it difficult to approach methanol by steric hindrance, resulting in a difficulty in both physical and electrostatic interaction among the components, which may contribute to the observed increase in surface tension.^6,17–19^

From a physicochemical consideration, the distinct behavior observed for the oil- and methanol-containing systems suggests that [DETA]­[Hx] may interact differently depending on the polarity of the surrounding medium. Such behavior is consistent with physicochemical trends reported for amphiphilic ionic liquid systems, in which simultaneous interactions with polar and apolar environments can influence phase-interaction behavior and interfacial organization in biphasic media.^6,14,19^ In the present study, the reduction in surface tension observed for the oil-containing system, combined with the altered behavior observed in the methanol-containing system, suggests that [DETA]­[Hx] may contribute to modifications in intermolecular interactions within the reaction medium under alkaline transesterification conditions. Although these effects were evaluated using simplified binary systems rather than the complete reactive ternary medium, the combined physicochemical and reaction performance results reinforce the relevance of further investigating low-concentration protic ionic liquids as physicochemical additives capable of influencing biphasic transesterification behavior.

The physicochemical interpretation proposed in the present study is consistent with previous observations that alkaline transesterification systems may initially operate under mass-transfer-controlled conditions due to the intrinsic immiscibility between methanol and triglyceride phases. Noureddini and Zhu^62^ reported that the early stages of soybean-oil methanolysis were strongly influenced by mixing intensity, proposing an initial transport-controlled regime, followed by a kinetically controlled reaction stage. Similarly, Stamenković et al.^63^ demonstrated that increases in agitation intensity reduced droplet size and increased interfacial area in methanol–oil systems, directly affecting methanolysis progression in biphasic media. In addition, Boocock et al.^64^ showed that the incorporation of cosolvents capable of generating pseudohomogeneous reaction environments substantially accelerated methanolysis reactions by reducing diffusional limitations associated with phase separation. These observations provide a relevant literature basis for contextualizing the physicochemical behavior observed for [DETA]­[Hx] under the evaluated conditions.

Although the observed surface tension behavior and biodiesel yield improvements are consistent with the hypothesis that [DETA]­[Hx] influences phase interactions within the reaction medium, these results should be interpreted as indirect physicochemical evidence rather than as direct confirmation of enhanced mass transfer or complete ionic liquid additive behavior throughout the transesterification process. A more comprehensive understanding of these effects would require a systematic investigation of compositional ratios, interfacial phenomena, and transport-related parameters under dynamic reaction conditions.

When compared with studies reported in the literature, the present system achieved relatively high biodiesel yields under comparatively mild operational conditions, including lower reaction times, lower methanol-to-oil molar ratios, and low ionic liquid concentrations. Table 7 summarizes representative biodiesel yields reported for ionic-liquid-based and conventional cosolvent-assisted transesterification systems.

**Table 7:** Comparison of Biodiesel Yields and Operating Conditions Obtained Using [DETA]­[Hx], Ionic Liquids, and Conventional Co-solvent Systems

category	additive	loading	yield (%)	T (°C)	time (min)	MeOH:Oil	ref.
**ionic liquid**	**[DETA][Hx]**	**3 wt %**	**97.1**	**60**	**30**	**9** **:** **1**	**this work**
ionic liquid	[Cho][Arg]	14.75 wt %	98.6	81.4	56.4	9.13:1	^24^
ionic liquid	DACARB (*N,N-diallylammonium N′,N′-diallylcarbamate*)	2 wt %	91.3	50	720	4:1	^30^
ionic liquid	BBAIL (*benzimidazolium-based Brønsted acid ionic liquid*)	4 wt %	96.0	50	90	10:1	^31^
ionic liquid	[DMIm]HSO_4_	0.1 g (for 2 mmol of fatty acid)	97.0	70	600	13.5:1	^33^
ionic liquid	[N_2222_][Arg]	20 wt %	98.4	100	60	10:1	^59^
ionic liquid	ChOH	2 wt %	95.0	65	30	10:1	^65^
ionic liquid	[P_4444_][For]	8 g^–1^ biomass	98.7	102.4	276	9:1	^66^
conventional cosolvent	THF	THF:MeOH = 1.4:1	97.0	52	30	6:1	^10^
conventional cosolvent	*n*-hexane	oil:hexane = 1:6	96.0	40	60	6:1	^12^
conventional cosolvent	acetone	20 wt %	98.0	55	25	6:1	^67^
conventional cosolvent	THF	10 vol %	98.5	65	90	12:1	^68^
conventional cosolvent	acetone	20 wt %	99.0	40	30	6:1	^69^
conventional cosolvent	acetone	25 wt %	98.0	25	30	4.5:1	^70^
conventional cosolvent	*n*-hexane	hexane:MeOH = 1.5:1	97.2–98.6	50	30	5:1	^71^

It is also noteworthy that several studies summarized in Table 7, including recent investigations involving ionic liquids and interfacial additives, primarily evaluated biodiesel production performance through mass-based yield or conversion measurements. For example, Lima et al.,^65^ Mascarenhas et al.,^5^ and Huang et al.^24^ employed mass-based performance indicators as the principal basis for comparing transesterification efficiency. In the present study, the reaction performance was specifically assessed by the gravimetric biodiesel yield. Although complementary compositional analyses, such as fatty acid methyl ester (FAME) profiling and residual glyceride determination, would provide a more comprehensive assessment of chemical conversion and biodiesel composition, the gravimetric yield remains a useful screening metric for comparing the effects of additives and operating conditions under controlled experimental conditions.

Despite employing only 3 wt % [DETA]­[Hx] under conventional alkaline transesterification conditions, the present study achieved biodiesel yields comparable to those reported for a wide range of ionic-liquid and conventional cosolvent systems. Notably, several of the highest yields reported in the literature were obtained using substantially higher ionic liquid loadings, elevated temperatures, longer reaction times, higher methanol-to-oil ratios, or process-intensification strategies such as ultrasound assistance. In contrast, the present system reached 97.06% biodiesel yield at 60 °C and a methanol-to-oil ratio of 9:1 without requiring auxiliary energy inputs or highly intensified operating conditions. Furthermore, while conventional cosolvents such as THF, acetone, and *n*-hexane often require relatively large solvent fractions to promote phase homogenization, comparable biodiesel yields were obtained in the present work using a considerably lower concentration of a protic ionic liquid. Although these comparisons highlight the competitive biodiesel production performance obtained with low concentrations of [DETA]­[Hx], practical implementation of ionic-liquid-assisted systems also depends on their recovery and reuse after reaction.

The practical applicability of protic ionic liquids in biodiesel transesterification is also influenced by their recovery and reuse. Their negligible vapor pressure, thermal stability, and tunable physicochemical properties may favor recovery from multiphase systems.^3,16,22^ Accordingly, reuse has been demonstrated in ionic-liquid-assisted processes, with Ibrahim et al.^26^ reporting biodiesel conversions above 95% after six reuse cycles of [NMP]­[HSO4], while sulfonated dicationic ionic liquid systems maintained yields above 90% after nine oleic acid esterification cycles.^72^ Structural analyses of recovered ionic liquids have also indicated preservation of their chemical characteristics during repeated use.^26,72^

High recovery efficiency remains important because ionic liquid losses can directly affect process feasibility, with efficiencies above 99% frequently considered desirable for large-scale ionic liquid-assisted processes.^18,22^ Recovery may also be affected by phase partitioning, accumulation of glycerol and reaction intermediates, and material losses during purification.^3,17^ These considerations highlight recovery behavior as an important aspect for evaluating the practical application of [DETA]­[Hx]-assisted transesterification.

### 3.5 Biodiesel Property Evaluation

Globally, to maintain the quality of the final product and to ensure adequate biodiesel storage and marketing criteria, some properties should be measured and must comply with current specifications. Therefore, biodiesel samples synthesized with 3% w/w of ionic liquid additive showed the highest biodiesel yield in 30 and 60 min of reaction and were analyzed in terms of appearance, specific gravity, kinematic viscosity, and flash point to check compliance with the requirements of European Standard EN 14214,^45^ American Standard ASTM D6751,^46^ and Brazilian Standard ANP 45/2014.^47^ Table 8 shows that the samples analyzed meet the specifications of European, American, and Brazilian standards.

**Table 8:** Physical–Chemical Properties of Samples of 3% w/w of [DETA]­[Hx] in 30 and 60 min of Reaction

property	biodiesel (30 min)	biodiesel (60 min)	ANP 45/2014	EN 14214	ASTM D6751	method
appearance	clear and free of impurities	clear and free of impurities	clear and free of impurities	-	-	-
density at 20 °C (kg·m^–3^)	883.4	884.2	850–900	860–900	-	ASTM D4052 ^37^
kinematic viscosity at 40 °C (mm^2^·s^–1^)	4.2085	4.3526	3.0–6.0	3.5–5.0	1.9–5.0	ASTM D445 ^38^
flash point (°C)	176	170	≥100	≥101	≥130	ASTM D93 ^44^

The physicochemical properties obtained for the biodiesel samples produced using 3% w/w [DETA]­[Hx] remained within the specifications established by ASTM D6751, EN 14214, and ANP 45/2014 standards. In particular, the density and kinematic viscosity values were consistent with those commonly reported for soybean biodiesel produced via conventional alkaline transesterification processes, indicating that the use of low concentrations of [DETA]­[Hx] did not adversely affect the fuel quality parameters evaluated in this study.

Furthermore, the high flash point values suggest effective methanol removal during purification, which is an important requirement for the biodiesel storage and handling safety. These results reinforce that the incorporation of [DETA]­[Hx] under the evaluated conditions was compatible with the production of biodiesel, meeting the principal international fuel quality specifications.

The fact that the evaluated fuel properties remained within the principal international specifications, even under low-concentration [DETA]­[Hx] conditions, reinforces the relevance of physicochemical additive strategies aimed at improving the transesterification performance without compromising biodiesel quality parameters.

## 4 Perspectives and Future Developments

The behavior observed for [DETA]­[Hx] under low-concentration conditions suggests that physicochemical and interfacial phenomena may represent an important aspect of the influence of protic ionic liquids on the alkaline transesterification systems. These findings reinforce the need for further investigations focused on how ionic liquid-induced modifications in phase interactions affect reaction performance in biphasic transesterification media.

On the basis of the present findings, the next stage of investigation should focus on directly correlating reaction performance with transport and interfacial phenomena. In particular, quantitative evaluation of parameters such as the volumetric mass transfer coefficient (kLa), interfacial area, droplet size distribution, and dispersion dynamics will be essential to determine whether the observed increases in biodiesel yield are effectively associated with reductions in diffusional limitations. The integration of these analyses with kinetic modeling may provide a more comprehensive understanding of the relative contributions of transport effects and reaction chemistry during the alkaline transesterification.

A deeper understanding of these systems will also require a systematic investigation of how the compositional relationships among protic ionic liquids, methanol, and oil phases influence the physicochemical behavior of the reaction medium. The physicochemical behavior observed in the present study, particularly the distinct surface tension responses obtained for the oil- and methanol-containing systems together with the measurable biodiesel yield improvements under low-concentration conditions, suggests that physicochemical effects associated with phase behavior and intermolecular interactions may represent a relevant aspect of protic ionic liquid behavior in alkaline transesterification systems. In this context, future investigations should systematically examine interfacial behavior within the complete reactive ternary system involving protic ionic liquids, methanol, and oil phases under dynamic transesterification conditions. Direct evaluation of interfacial tension, phase-dispersion behavior, and composition-dependent physicochemical interactions in the reactive medium may provide a more comprehensive understanding of whether the observed reaction performance improvements are associated with modifications in interfacial transport phenomena.

In particular, interfacial physicochemical properties, pH behavior, phase dispersion characteristics, and local physicochemical conditions within the biphasic system should be evaluated under the varying operational conditions. Expanded experimental designs incorporating ionic liquid-to-methanol ratios may therefore provide a more comprehensive understanding of how protic ionic liquids influence phase interaction behavior and reaction environment during biodiesel production.

The low concentrations of [DETA]­[Hx] required to produce measurable effects suggest that future investigations should prioritize process simplification strategies rather than approaches based on increasing the ionic liquid loading. In this context, comparative studies involving conventional cosolvents and protic ionic liquids under equivalent operational conditions will be particularly important to establish whether the advantages associated with ionic liquids arise predominantly from interfacial behavior, solvent effects, or combined physicochemical phenomena.

From a technological perspective, subsequent studies should also address ionic liquid recovery, recyclability, and partition behavior during washing and purification stages since these aspects will directly determine the practical applicability of ionic-liquid-assisted transesterification systems. Likewise, detailed compositional analyses of the biodiesel phase, including FAME profile determination and residual glyceride quantification, will be important to establishing possible relationships between physicochemical effects and fuel quality.

Overall, the present work establishes an initial experimental basis for investigating protic ionic liquids as low-concentration physicochemical additives in biodiesel production while also indicating that future advances in this field will depend on the integration of reaction engineering, transport phenomena, and interfacial characterization approaches.

## 5 Conclusion

This study demonstrated that diethylenetriammonium hexanoate ([DETA]­[Hx]) can effectively influence the performance of conventional KOH-catalyzed soybean oil transesterification when employed as a low-concentration physicochemical additive. Within the investigated experimental domain, [DETA]­[Hx] concentration was the significant factor affecting gravimetric biodiesel yield, and increasing its concentration from 0 to 3% (w/w) increased the yield from 92.0% to 97.1% at 30 min, corresponding to a gain of 5.1 percentage points. This performance was achieved at 60 °C, with a methanol-to-oil molar ratio of 9:1 and 1.5% (w/w) KOH. In addition, the evaluated density, kinematic viscosity, appearance, and flash point of the resulting biodiesel remained within the applicable ASTM D6751, EN 14214, and ANP specifications.

The reaction-performance results were consistent with the physicochemical evidence obtained for [DETA]­[Hx]. Its surface-active characteristics, the distinct surface-tension responses observed in the oil- and methanol-containing systems, and the preservation of its principal vibrational features in the [DETA]­[Hx]–KOH–methanol system collectively support a physicochemical role in the biphasic reaction medium rather than direct catalytic participation. Accordingly, the observed improvement is consistent with a physicochemical influence of [DETA]­[Hx] on the reaction environment.

The main contribution of this work therefore lies in demonstrating that [DETA]­[Hx], a protic ionic liquid not previously investigated for this application, can be employed at a concentration of only 3% (w/w) as a physicochemical additive in a KOH-catalyzed transesterification system, extending the use of ionic liquids beyond their more conventional roles as catalysts or bulk reaction media. From an application perspective, the preliminary mass-balance-based sensitivity analysis indicated that the practical relevance of the observed yield improvement depends on the [DETA]­[Hx] cost and recovery, providing an initial quantitative perspective on these relationships without constituting a techno-economic assessment of the process. These findings establish an experimental basis for the use of low-concentration PILs as physicochemical modifiers in biphasic biodiesel-production systems.

## Notes

The authors used ChatGPT (OpenAI) solely for English language editing, translation, figure quality enhancement, and the creation of the graphical abstract. All outputs were reviewed and verified by the authors, who take full responsibility for the final manuscript.
